# Role of reactive oxygen species in ultraviolet-induced photodamage of the skin

**DOI:** 10.1186/s13008-024-00107-z

**Published:** 2024-01-12

**Authors:** Min Wei, Xin He, Na Liu, Hui Deng

**Affiliations:** 1 Department of Dermatology, Shanghai Sixth People’s Hospital Affiliated to Shanghai Jiao Tong University School of Medicine, Shanghai, China; 2 Department of Nephrology, Beijing Chao-Yang Hospital, Capital Medical University, Beijing, China

**Keywords:** Reactive oxygen species, Ultraviolet, Photodamage, DNA, Inflammation, Apoptosis

## Abstract

Reactive oxygen species (ROS), such as superoxides (O_2_ •−) and hydroxyl groups (OH·), are short-lived molecules containing unpaired electrons. Intracellular ROS are believed to be mainly produced by the mitochondria and NADPH oxidase (NOX) and can be associated with various physiological processes, such as proliferation, cell signaling, and oxygen homeostasis. In recent years, many studies have indicated that ROS play crucial roles in regulating ultraviolet (UV)-induced photodamage of the skin, including exogenous aging, which accounts for 80% of aging. However, to the best of our knowledge, the detailed signaling pathways, especially those related to the mechanisms underlying apoptosis in which ROS are involved have not been reviewed previously. In this review, we elaborate on the biological characteristics of ROS and its role in regulating UV-induced photodamage of the skin.

## Introduction

**Fig. 1:**
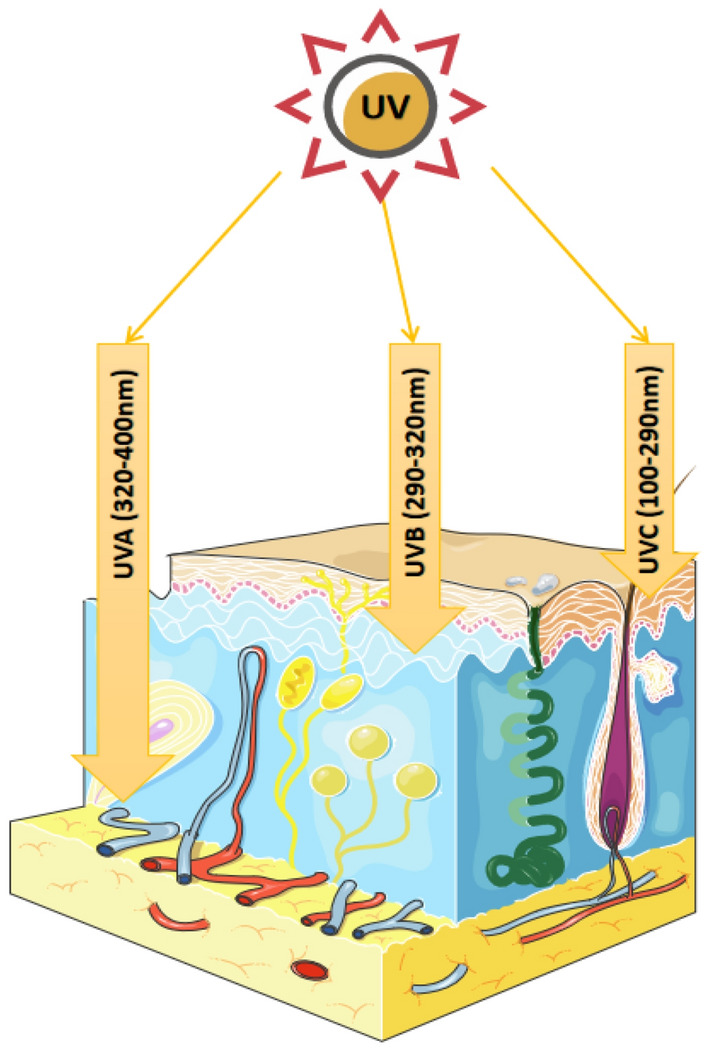
Depths of ultraviolet (UV) radiation (UVR). UVC, with the shortest wavelength (100–290 nm) and highest energy, is mostly blocked by the ozone layer and rarely reaches human skin. Contrastingly, both UVB and UVA can penetrate the ozone layer. UVA, with the longest wavelength (320–400 nm) and lowest energy, can reach deep into the dermis, whereas UVB (wavelength: 290–320 nm) mostly reaches the surface of the dermis

The ultraviolet (UV) component of sunlight can be divided into ultraviolet C (UVC; wavelength: 100–290 nm), ultraviolet B (UVB; wavelength: 290–320 nm), and ultraviolet A (UVA; wavelength: 320–400 nm), according to their different wavelengths [1]. UVC is mostly blocked by the ozone layer and rarely reaches the human skin, while both UVB and UVA can penetrate the ozone layer, accounting for about 95% and 5% of UV radiation (UVR) that reaches the skin, respectively [2] (Fig. 1). UVR can contribute to dermal photodamage via DNA damage, inflammation, oxidative stress, and apoptosis, leading to external signs of skin damage. For example, exogenous aging, which accounts for 80% of skin aging, manifests with the development of wrinkles, skin relaxation, hyperpigmentation [3].

In recent years, many studies have indicated that reactive oxygen species (ROS), which are molecules with short lives that compromised of unpaired electrons [4], play crucial roles in regulating UV-induced photodamage of the skin. Hence, to better address the harmful effects of UV-induced photodamage, understanding how it is regulated by ROS is crucial. In this review, we elaborate and focus on the biological characteristics of ROS and its role in regulating UV-induced photodamage of the skin.

## Reactive oxygen species

**Fig. 2:**
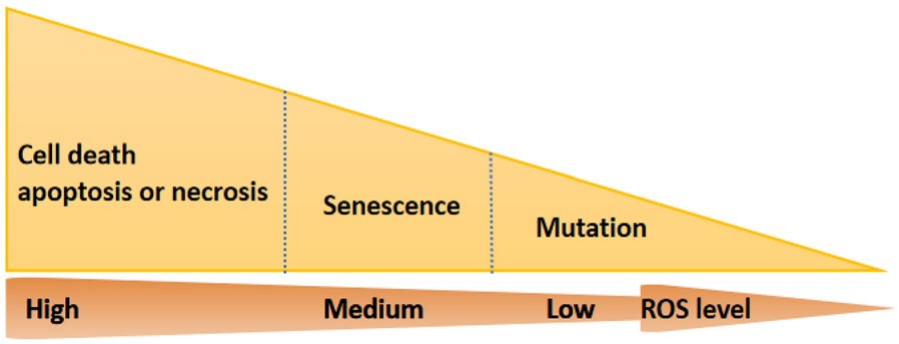
Effects of reactive oxygen species (ROS) depend on the dose and persistence of the particles. A low level of ROS can lead to mutation, a medium level of ROS can cause senescence, and a high level of ROS usually cause cell death, such as apoptosis and necrosis

ROS, which are the molecules containing highly unstable oxygen radicals, such as superoxide (O_2_ •−) and hydroxyl group (OH•), usually can be rapidly transformed to more stable non-radicals like hydrogen peroxide (H_2_O_2_) and hypochlorous acid, which can diffuse easily [4] [5]. Hence, ROS can oxidize sulfhydryl groups with cysteine residues, including proteins such as kinases, phosphatases and transcription factors [6]. They can also participate in physiological processes, such as cell signaling, proliferation, and tumor suppression, supporting the immune system for pathogen resistance and oxygen homeostasis [7]. The effects of ROS depend on the dose and persistence of the ROS particles, as well as the type of cells involved. A low level of ROS can lead to mutation, a medium level of ROS can cause senescence, and a high level of ROS usually lead to cell death, such as apoptosis or necrosis [8] (Fig. 2).

### ROS production

Intracellular ROS can be produced by various systems, including mitochondrial electron transport chain (ETC), NADPH oxidase (NOX), cytochrome P450, lipoxygenase, xanthine oxidase, nitric oxide synthase, and cyclooxygenase [9].

**Fig. 3:**
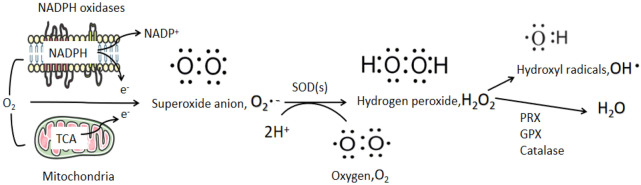
Production and regulation of reactive oxygen species (ROS). Mitochondria and NADPH oxidases (NOXs) are the two main contributors of endogenous ROS. O_2_• − is formed from molecular O_2_ following the acceptance of a single electron from the electron transport chain (ETC) in the mitochondria or from NOXs. Superoxide dismutase (SOD) enzymes convert O_2_ •− into H_2_O_2_, which can then be reduced and converted by peroxiredoxin (PRX), glutathione peroxidase (GPX), and catalase to form H_2_O and OH•. The latter is extremely reactive and causes damage to DNA, proteins, and lipids

Large part of endogenous ROS are produced from mitochondria, which contain 10 sites that are known to be capable of producing O_2_ •− , and 1% of the consumed O_2_ is used to produce O_2_ •− [10]. Mitochondria produce ATP via the oxidation process of glucose, amino acids, and lipids. The Krebs cycle involves removing an electron from these metabolites and transferring it to the ETC, thereby reducing O_2_ to O_2_ •− [11]. The energy released by the transport of electrons is utilized to expel protons (H +) from the mitochondrial matrix across the mitochondrial inner membrane (at multisubunit protein complexes include complex I, complex III, and complex IV) into the intermembrane space [12]. And O_2_ •− can be released both into the intermembrane space and mitochondrial matrix by complex III, involving in many biological processes [13]. Meanwhile, most of the O_2_ •− produced by mitochondria are changed to H_2_O_2_ by manganese-superoxide dismutase in the mitochondrial matrix [14]. With the facility of specific members of the aquaporin family, H_2_O_2_ can be highly diffused through the mitochondrial membrane [15]. Members of the NOX family are considered to have the primary function of producing ROS and serve as the primary source of ROS [16]. The NOX family contains seven isomers, which share similar molecular structure and function, are all transmembrane proteins. It includes NOX1–5 and dual oxidases 1 and 2, with one NADPH-binding site, one flavin adenine dinucleotide (FAD)-binding site, six transmembrane domains, and four heme-binding histidines [17]. NOX transmits electrons through biofilms to produce O_2_ •− , which is also transformed to H_2_O_2_ quickly [17] (Fig. 3).

### ROS removal

The homeostasis of ROS is important for cell survival and cell signaling of prevention of cell damage. The involvements of non-enzymatic or enzymatic antioxidants facilitate elimination of different types of ROS to achieve ROS detoxification, which contribute to the homeostasis of ROS. The most important antioxidant enzyme is glutathione peroxidase(GPX), which can regulate intracellular H_2_O_2_ level, maintain reduced and oxidized glutathione (GSH/GSSG) balance, and promote antioxidant enzyme activity by allowing sulfhydryl reaction with glutathione (GSH) to remove singlet oxygen, hydrogen peroxide, and organic peroxides, thus regulating ROS homeostasis. Enzymatic antioxidants include superoxide dismutase (SOD), catalase, GSH, peroxiredoxin(PRX), and thioredoxin [18]. SOD enzymes can dismutate O_2_ •− into H_2_O_2_, which then be reduced and converted to form H_2_O and OH•, an extremely reactive free radical that can damage DNA, proteins, and lipids. Non-enzymatic antioxidants include GSH; flavonoids; vitamins A, C, and E; and ubiquinone [19]. Antioxidants play are key in degrading O_2_ •− and H_2_O_2_, thus reducing the damage of oxidation (Fig. 3).

## Role of ROS in UV-induced photodamage of the skin

Normal skin tissue mainly includes keratinocytes in epidermis, melanocytes near the basement membrane, fibroblasts in dermis and some extracellular matrix. The morphology of the aged skin is aging and atrophy, with a decrease in the thickness of epidermis and dermis, a decrease in the composition of collagen and elastin in extracellular matrix, and a decrease in the total number of fibroblasts [20]. Photoaged skin usually presents with proliferation of skin cells, and increased thickness of stratum corneum, epidermis and dermis. Photoaged epidermis is characterized by acanthosis and excessive keratinization, and the clinical manifestation is rough and dry skin. Fibroblasts and inflammatory cells were increased in the photoaged dermis. The distribution of melanocytes is not uniform, resulting in pigmentation and pigmentation spots. The amount of interstitial collagen in photoaging is decreased and damaged, leading to the disorder of skin molecular structure [21].

**Fig. 4:**
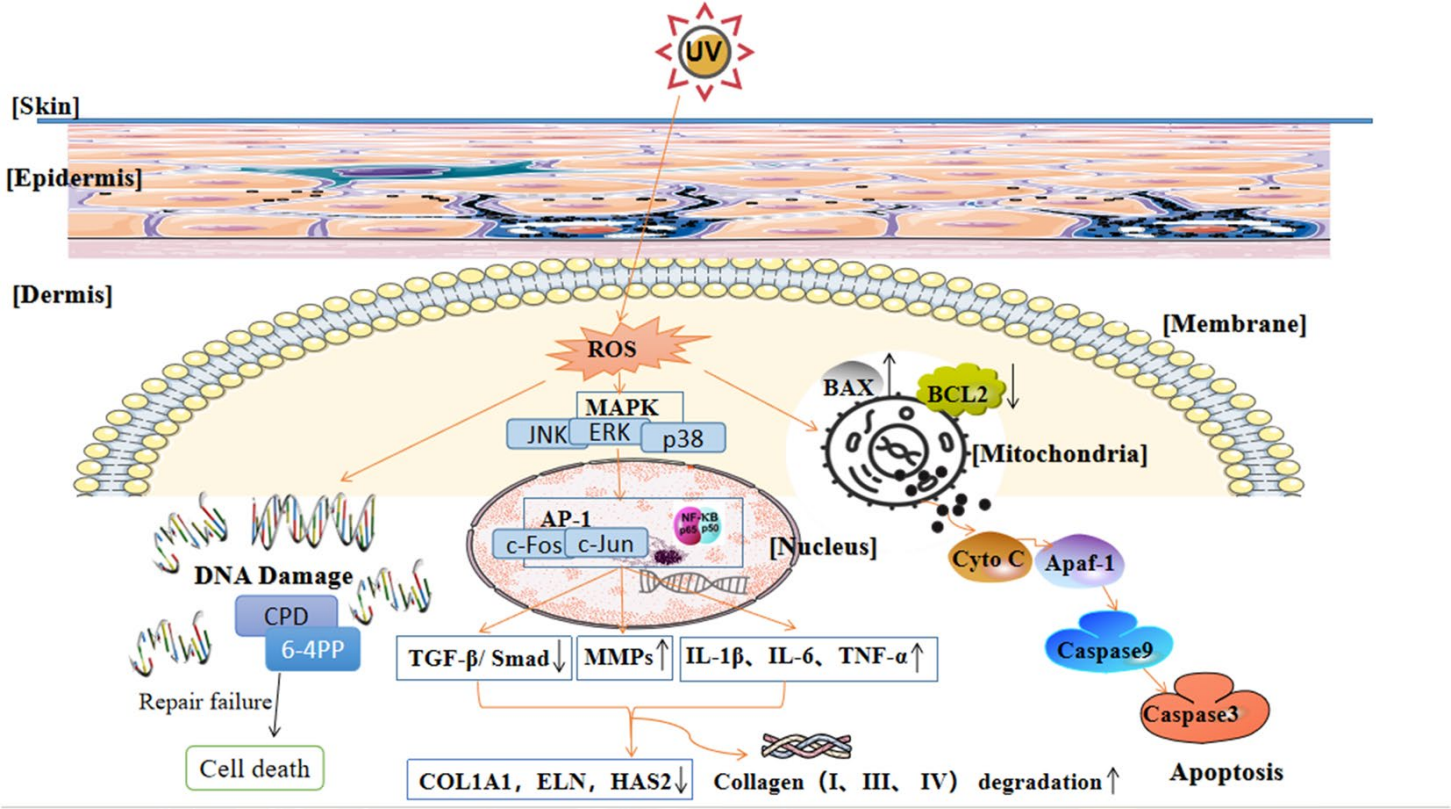
Signaling pathways of reactive oxygen species (ROS) in ultraviolet (UV)-induced skin photodamage. ROS regulate DNA damage and cell signaling pathways, and cause an imbalance of skin oxidants and antioxidants, which accelerates skin photodamage. Unrepaired damaged DNA, including cyclobutane pyrimidine dimers (CPDs) and pyrimidine (6–4) pyrimidone photoproducts [(6–4)PPs], can cause cell death. Moreover, ROS signaling regulates transcription factors, such as AP-1 and nuclear factor-kappa B (NF-κB), inducing the expression of matrix metalloproteinases (MMPs), upregulating the expression of inflammatory factors, such as IL-1β, IL-6, and tumor necrosis factor (TNF)-α, and downregulating TGF-β expression and Smad signaling. Thus, extracellular matrix genes and collagen type I alpha1 (COL1A1), elastin (ELN), and hyaluronan synthase 2 (HAS2) are downregulated. Moreover, collagen types I and III are degraded into ¾ and ¼ fragments, respectively, while type IV collagen is degraded into non-collagen components and elastic fibers, resulting in accelerated aging in the form of skin relaxation, deepened wrinkles, and decreased skin elasticity. ROS are also directly or indirectly involved in UV-induced mitochondrial apoptosis by affecting the expression of Bcl-2 and Bcl-x in the mitochondria, thus inducing the release of cytochrome c (cyt-c). Once released, cyt-c functions with the apoptotic peptidase activating factor 1 (Apaf-1) to form a type of apoptotic body that recruits and activates caspase 9 to initiate caspase 3-dependent apoptosis

Long-term exposure to UV can cause DNA damage, oxidative stress, inflammation, and cell apoptosis [22]. UV can interact with chromogenic groups and photosensitizers in cells to produce ROS, such as superoxide anion radical and hydroxyl radical [23]. ROS may act as second messengers to regulate phosphorylation of multiple proteins in signal transduction pathways [24]. While the exact mechanism of photodamage is still being studied, it has been reported that increased ROS production, collagen degradation, and mitochondrial DNA damage are its key features. ROS can regulate DNA damage and cell signaling pathways, leading to an imbalance of skin antioxidants, thus accelerating skin photodamage [25]. ROS signaling regulates transcription factors, such as AP-1 and nuclear factor-kappa B (NF-κB), inducing the expression of matrix metalloproteinases (MMPs) that can induce collagen oxidation and reduce the expression of types I and III collagen [26]. In addition, ROS can regulate collagen metabolism, resulting in skin relaxation, deepening of wrinkles, and decreased skin elasticity [26]. Moreover, ROS are directly or indirectly involved in UV-induced mitochondrial apoptosis (Fig. 4).

**Fig. 5:**
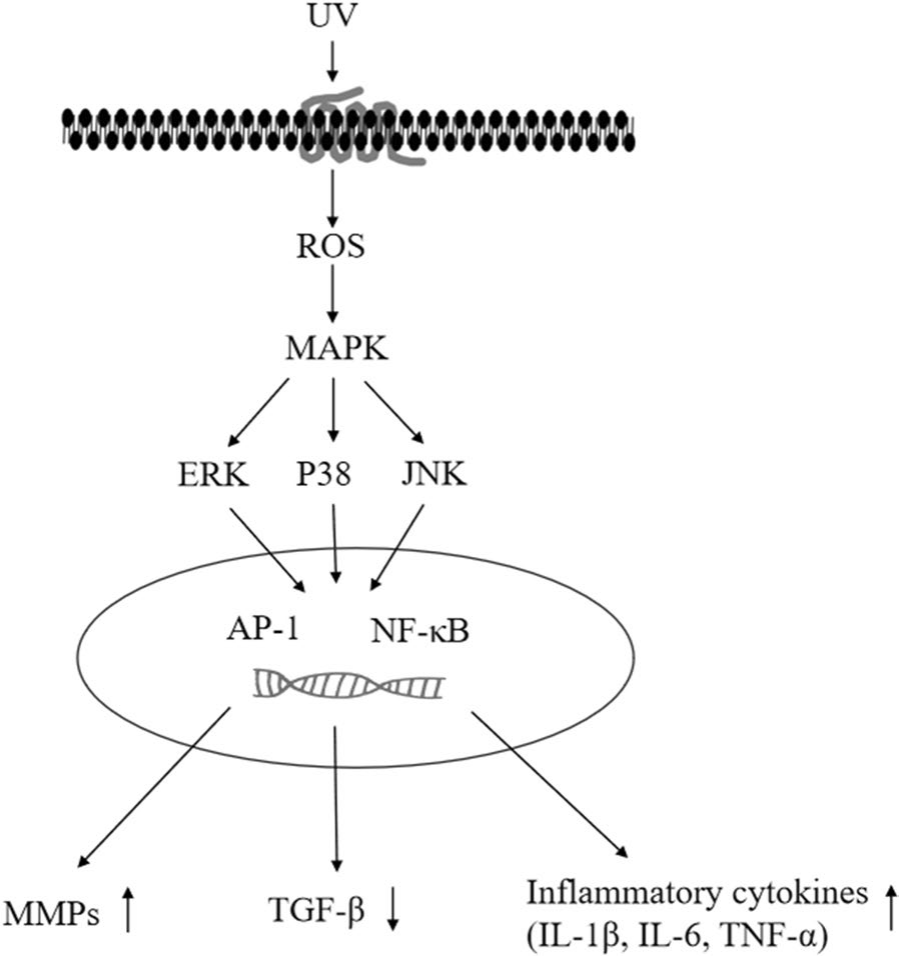
Signaling pathway of MAPK in UV-induced skin photodamage affected by ROS. UV irradiation can induce ROS and then has an impact on activating the MAPK pathway within the cells. Then, the downstream factors extracellular signal-regulated kinase (ERK), P38 kinase, and c-Jun N-terminal kinase (JNK) in the MAPK pathway can enter the nucleus and regulate transcription factors, such as AP-1 and NF-κB, inducing the expression of MMPs, upregulating the expression of inflammatory factors, such as IL-1β, IL-6, TNF-α, and downregulating TGF-β expression

In addition, ROS produced by ultraviolet radiation can also affect intracellular DNA through MAPK-induced signaling. Studies have found that ROS generated by ultraviolet radiation can not only regulate MAPK signaling pathway, but also simultaneously regulate signaling pathways such as JAK/STAT and extracellular signal-regulated kinase (ERK) [27, 28]. Further studies have found in UV-mediated inflammation, ROS activates MAPK pathway and downstream factors NF-κB and AP-1, thus can regulate the release of inflammatory cytokines such as IL-1β, IL-6 and TNF-α [29]. P38 and c-Jun N-terminal kinase (JNK) are two important downstream factors in MAPK signaling pathway [30]. After the activation of p38 and JNK in MAPK pathway, they can also enter the nucleus and upregulate the expression of related inflammatory cytokines, induce the expression of MMPs and downregulate TGF-β expression, thus playing an important role in the UV-mediated photodamage [31, 32] (Fig. 5).

### DNA damage by ROS

Both UVA and UVB can cause photodamage by inducing ROS production and oxidative damage [33], and ROS can directly or indirectly result in DNA damage.

The DNA damage caused by UV mainly affects the electronic structure of DNA by promoting chemical reactions between bases, especially the dimerization of thymine (cyclobutane derivative) [2]. The absorption of UV irradiation directly by DNA can result in adducts between adjacent pyrimidine nucleotides, forming cyclobutane pyrimidine dimers (CPDs) and pyrimidine (6–4)pyrimidone photoproducts [(6–4)PPs] [34]. 6-4PPs are larger and more massive in size, but more efficiently repaired than CPDs [35]. However, with larger numbers and slower repair rate, CPDs have more obvious mutagenicity than [(6–4)PPs] [36]. In addition, these products can hinder DNA and RNA from replicating, as a result of acting as physical obstacles to DNA polymerase and RNA polymerase and facilitating replication fork stagnation formation, leading to chain breakage that can consequently lead to chromosomal abnormalities[37] [38]. UVA-induced photoproducts, of which CPDs are the main products and [(6–4)PP] formation is not significant, are much less than those induced by UVB and were previously considered harmless in many cases [39]. UVB is considered to be the main reason of skin photoaging [40], with [(6–4)PPs] as its main products that trigger cell signaling pathways, activate the defense system, and lead to DNA repair or apoptosis [41]. Reportedly, ROS participate in stem cell self-renewal, which in response to DNA double-strand breaks, by actiating the ataxia-telangiectasia mutated protein kinase [42].

P53, a transcription factor that can activate the inhibitor of the cell cycle and participate in DNA damage response and transcription of apoptosis [43], may also play a role in premature aging by causing reactive damage to DNA [44]. p53 is associated with the two key kinases of ataxia telangiectasia and rad3-related protein-checkpoint kinase 1 pathway [45] that induce the transcription of cell cycle suppressor p21 gene and delay damaged G1 cells from entering S phase, preventing new start events at the beginning of the duplication process and slowing down the branching processes of reproduction of UV-irradiated cells in the S phase. Thus, p53 transiently suppresses DNA synthesis in UV-damaged cells [46]. Consequently, DNA repair can be completed before DNA synthesis to reduce the DNA mutation rate [47]. Studies have confirmed the involvement of the ROS-p38-p53 pathway in UV-induced cell damage, in which p38 mediates p53 phosphorylation after UV irradiation [48]. Moreover, ROS can affect the overexpression of p53 and p21^cip1/waf1^, leading to the failure of DNA or apoptosis repair [49].

### Mitochondrial DNA damage and apoptosis by ROS

Apoptosis is generally induced by the activation of cell surface death receptors (exogenous pathways) that are initiated mainly by the binding of death ligands of the TNF superfamily of cytokines, including TNF, Fas-ligand, and TNF-related apoptosis-inducing ligand with their death receptors TNF receptor 1, Fas (also known as CD95), and DR4 or DR5, respectively. It can also be induced by various signals from the mitochondria and endoplasmic reticulum (endogenous pathways) [50].

UV-induced apoptosis represents a clearing mechanism that eliminates DNA-damaged cells, thereby reducing the risk of malignant transformation. To sum up, ROS can be directly involved in UV-induced apoptosis [51] by not only destroying several key structural and functional proteins and DNA but also inducing the release of cytochrome c (cyt-c) from the mitochondria, thereby accelerating photodamage of the skin [52].

#### Apoptosis induced by unrepaired DNA damage

ROS can induce DNA damage, which appears to be a determinant of UV-induced apoptosis [51]. Obstruction of cell cycles provides time for the recognition and repair of UV photoproducts, most of which are removed by DNA excisional repair [53]. However, if DNA damages are extensive and cannot be repaired, and RNA polymerase cannot transcribe the necessary gene products, then apoptosis will be triggered [54].

#### Mitochondrial DNA damage and endogenous apoptosis

Large-scale mitochondrial DNA (mtDNA) deletion has been documented in skin tissues exposed to sunlight [55]. mtDNA damage also leads to the maladjustment of oxidative phosphorylation and an increase in ROS production [56]. The activation or inhibition of apoptosis is documented mainly determined by the balance between members of the Bax protein family, for example, pro-apoptotic proteins like Bax, Bak and Bid, and anti-apoptotic members such as Bcl-2 and Bcl-x [57]. Bcl-2 inhibits the activation of caspases 3 and 8 and promotes the release of cyt-c, while Bcl-x partially inhibits such release [58]. UV can promote the translocation of Bax to the mitochondria [59], increasing the expression of p53 and Bax, and decreasing the expression of Bcl-2 [60]. A study showed that ROS can affect the transcriptional regulation of the Bax gene to a large extent through p53 [61]. Once released in the cytoplasm, cyt-c works with the apoptotic peptidase activating factor 1 (Apaf-1) to form a kind of apoptotic body that recruits and activates caspase 9 to initiate caspase 3-dependent cell death [62].

Studies on nucleotide excision repair-defective cells have shown that unrepaired UV-induced DNA damage can trigger the degradation of Bcl-2, the activation of caspase 3, and the destruction of mitochondrial membrane potential; thus, leading to the apoptosis of cells [63]. Within 24 h of UV exposure, Bcl-2 protein levels drop by about 90%, and this decrease can be prevented by the treatment with protease inhibitors MG115 or MG132, or partially eliminated by caspase 3 inhibitor DEVD-FMK and caspase 9 inhibitor LEHD-FMK [64].

### Inflammation by ROS and its regulatory mechanism in skin connective tissue

Antioxidant enzymes in the skin that remove ROS can be depleted by prolonged exposure to UV light. Photooxidation activates mitogen-activated protein kinase (MAPK), and regulates the expression of NF-κB and activator protein-1 (AP-1) by increasing ROS [65]. AP-1 and NF-κB activate MMPs in the dermis and epidermis [66]. This reduces the synthesis of types I and III procollagen, thereby damaging the formation of new collagen while also degrading it; thus leading to photodamage [67]. Moreover, ROS can directly trigger collagen fiber collapse through the overexpression of MMP-1 [68].

MAPK is comprised of the ERK, JNK, and P38 kinase, variously involving in cell proliferation, apoptosis, and inflammation [52]. Blocking the MAPK pathway can inhibit the expression of pro-inflammatory cytokines, such as IL-6, Cyclooxygenase-2 (COX-2), and IL-8 [69].

The NF-κB pathway has been widely demonstrated to play a crucial role in mediating skin inflammation, and its activation is crucial to the aging process [70]. When activated, pro-inflammatory factors infiltrate into the skin, leading to clinical syndromes, such as erythema and epidermal hyperplasia [71]. Inactivated NF-κB is present as a cytoplasmic heterodimer, which consists of the P65 and P50 subunits, and can bind to the inhibitory protein IκB. IKK complex can be recruited by the ubiquitin binding protein NF-κB essential modulator (NEMO) on the new ubiquitin chain formed by the receptor. When activated, this NEMO-dependent IKK complex phosphorylates IκB at Ser32 and Ser36, and induces the ubiquitination of protein and the degradation of the proteasome, releasing the inhibitory effect of IκB on NF-κB [72]. Next, the activated NF-κB heterodimer translocates to the nucleus, promoting the transcription of pro-inflammatory genes [73], particularly, the genes that control apoptosis, causing the production of cytokines and interferons that regulate inflammatory responses. These targets include TNF-α, IL-1, IL-6, and IL-8 [74]. IKK also directly phosphorylates the p65/50 dimer at the position 536 of p65, which has been shown to further increase the nuclear transcriptional activity of NF-κB [75].

Photoaging is characterized by matrix metalloproteinase induction and dermal collagen loss [35]. The loss of skin elasticity and the reduction in collagen content leads to the formation of wrinkles [76], since collagen makes up about 70% of the dermis. Regulation of collagen is achieved by promoting the synthesis of cytokine transforming growth factor beta (TGF-β), inhibiting transcription factor AP-1, and activating the degradation of MMPs [68].

ROS can induce AP-1, a heterodimer composed of c-fos and c-jun, which downregulates type I collagen and upregulates MMPs in aging skin [77], and can also inhibit the TGF-β signaling pathway in skin fibroblasts. As a result, reducing synthesis of new collagen and decreasing collagen numbers in the dermis [78].

In human dermal fibroblasts, TGF-β/Smad signaling pathway is key to maintain the integrity of the dermal structure, by increasing the extracellular matrix (ECM) production, and decreasing its degradation [79]. Initially, TGF-β binds to TGF-β receptor II (TβRII), which then recruits and phosphorylates TGF-β receptor I (TβRI), leading to the activation of transcription factors Smad2 and Smad3. And then the activated Smad2 or Smad3 bind to Smad4, forming the heterogeneous Smad complex, which translocates into the nucleus and interacts with Smad-binding element (SBE) of the TGF-β target gene [80]. Therefore, TGF-β/ Smad signaling directly upregulates ECM genes, including those involved in the production of collagen, fibronectin, decorin, and versican [78].

MMPs, which are zinc-dependent enzymes, play key roles in the degradation of collagen in dermal cells. To date, there has been 28 MMPs are identified [81]. Oxidative stress is involved in the degradation of collagen, while inflammation stimulates epidermal thickness, and these two interact with each other in both processes. The expression and activation of MMP-1, MMP-2, MMP-3, MMP-9 and/or MMP-13 are increased by oxidative stress, thereby reducing the collagens in the skin [82]. Among them, MMP-1 and -3 are important mediators for the degradation of ECM and the formation of skin wrinkles during UVB-induced photoaging [83]. MMP-1 (collagenase type I) is a fully functional mesenchymal collagenase-1 that can initiate the degradation of collagenase types I and III into ¾ and ¼ fragments, respectively [84]. Moreover, MMP-3 degrades type IV collagen into non-collagen components and elastic fibers. Overexpression of MMP-1 and MMP-3 are widely believed to result in wrinkle formation and sagging skin [72]. MMP-2 (gelatinase-A) and MMP-9 (gelatinase-B) degrade gelatin. MMP-2, MMP-7 and MMP-12 (elastase) degrade elastin [69]. Upregulated MMPs inhibit collagen type I alpha1 (COL1A1), elastin (ELN), and hyaluronan synthase 2 (HAS2), and regulates the synthesis of hyaluronic acid [69].

Recent studies have found that inflammasomes play an important role in UV-induced skin photodamage. Inflammasomes mainly include Nucleotide-binding domain and leucine-rich repeat pyrin-domain containing protein 1 (NLRP1) and NLRP3. And ROS induced by photodamge of the skin can activate inflammasomes. Studies have found that NLRP1 inflammasomes have taken centre stage in skin biology, as mutations in NLRP1 underlie the genetic etiology of dermatological diseases and increase the susceptibility to skin cancer [85]. Other studies have found that the activation of NLRP3 can recruit and activate pro-inflammatory protease caspase-1, and then produce IL-1β, IL-18 and other cytokines, triggering a process of inflammation-relatedcell death named pyroptosis. Indeed, pyroptosis is a rapid and inflammatory form of lytic programmed cell death [86]. Therefore, inflammasomes can be considered significant in UV-induced skin photodamage and may be potential targets for the treatment and improvement of photodamge of the skin.

## Conclusion

UV has been discovered as the main reason for the induction of photodamage, and there are many mechanisms involved in UV-induced photodamage. ROS, as key signaling molecules of life, play an increasingly central role in UV-induced photodamage, in which, elevated ROS levels have been documented. However, to the best of our knowledge, the detailed signaling pathways, especially the apoptosis mechanisms in which ROS are involved have not been reviewed previously. Hence, in this review, we discussed ROS, UV, and UV-induced photodamage. Moreover, the associated signal transduction of ROS in UV-induced photodamage has been summarized. ROS play a crucial role in inflammation, activating the MAPK signaling pathway, along with NF-κB and AP-1, which leads to the release of inflammatory factors, such as IL-1β, IL-6 and TNF-α; upregulation of MMPs, and degradation of collagen fibers. Consequently, the photoaged skin exhibits clinical symptoms, such as inflammatory erythema, skin relaxation, and wrinkles. Furthermore, ROS are involved in DNA damage, including mtDNA damage, and induces mitochondrial apoptosis. They can also disrupt the balance between members of the Bax protein family, accelerating cell death, which is also closely related to the pathogenesis of skin cancer. The information highlighted in this review will be helpful to future research and development programs of relevant antioxidants and can contribute to the discovery of targets that can reduce or prevent photodamage. Moreover, since many anticancer drugs destroy DNA in a similar manner to UVR [64], further research into the mechanisms underlying UV damage could also contribute to the understanding of the mechanisms of action of these highly relevant drugs.

## Data Availability

This is a review article, no data associated in this manuscript.
